# Publication Trends for Sarcopenia in the World: A 20-Year Bibliometric Analysis

**DOI:** 10.3389/fmed.2022.802651

**Published:** 2022-02-11

**Authors:** Dongliang Yuan, Hongfu Jin, Qianqi Liu, Jinglve Zhang, Boyan Ma, Wenfeng Xiao, Yusheng Li

**Affiliations:** ^1^Department of Orthopedics, Xiangya Hospital, Central South University, Changsha, China; ^2^Xiangya School of Medicine, Central South University, Changsha, China; ^3^National Clinical Research Center for Geriatric Disorders, Xiangya Hospital, Central South University, Changsha, China

**Keywords:** bibliometric analysis, sarcopenia, Web of Science, PubMed, publication

## Abstract

**Background:**

Sarcopenia, an age-related degenerative disease, seriously affects the health and quality of life of the elder. The research of sarcopenia has changed dramatically around the world. This article aims to analyze global trends in this field over the past 20 years.

**Methods:**

“Sarcopenia” was used as the search term to retrieve relevant publications from the WOS and PubMed databases. Co-occurrence, literature coupling, co-citation, and co-author analysis were performed by using the software VOS viewer. We analyzed the trends of sarcopenia research over the last 20 years from different aspects, such as the number of papers, total citations, average citations per item, h-index, research area, article types, institutions, country, journals, and funding.

**Results:**

We retrieved 13,421 research articles published on sarcopenia between 2001 and 2020. The results showed that the USA made the highest contributions to this field. Geriatrics gerontology is the most study classification of sarcopenia. Basic research on sarcopenia in geriatric gerontology accounts for approximately 16.496% of global publications. The Osteoporosis International published the largest number of sarcopenia-related studies. The United States Department of Health Human Services was the leading funding organization, which sponsored 1,604 articles.

**Conclusion:**

Global sarcopenia research increased rapidly from 2001 to 2020, especially recently. The research leader of sarcopenia is the USA. In the future, the study of sarcopenia will continue to focus on aging, nutrition, and exercise and will delve deeper into molecular mechanisms. On the other hand, revealing the link between sarcopenia and other diseases will be the next research hotspot.

## Introduction

Age-related decline in skeletal muscle mass was first defined as sarcopenia by Irwin Rosenberg in 1989, which is associated with an increased risk, such as physical disability, fracture caused by fall, and so on (1, 2). In addition to low muscle mass, the presence of low muscle function has also received attention. The European Working Group on Sarcopenia in Older People (EWGSOP) recommended using declined muscle mass and poor physical performance to diagnose sarcopenia in 2010, and they also issued an updated consensus in 2019, which presents details of the criteria recommended by the EWGSOP for the diagnosis of sarcopenia (3, 4) (Tables 1, 2). The Asian Working Group for Sarcopenia (AWGS) also published the sarcopenia consensus in 2014 and published an updated version in 2019. The recommendations of the consensus meeting of the Society of Sarcopenia, Cachexia and Wasting Disorders (SCWD) in 2019 for sarcopenia using the simple five-item questionnaire (SARC-F) questionnaire, grip strength or chair stance, and combined use of Dual X-ray Absorptiometry (DXA) of limb muscle mass to achieve rapid screening of sarcopenia (5). All these showed the rapid development of clinical and research interest in sarcopenia internationally.

**Table 1:** Criteria for the diagnosis of sarcopenia (2010).

**Diagnosis is based on documentation of criterion 1 plus (criterion 2 or criterion 3)**
1. Low muscle mass 2. Low muscle strength 3. Low physical performance

**Table 2:** Operational definition of sarcopenia (2019).

Probable sarcopenia is identified by Criterion 1. Diagnosis is confirmed by additional documentation of Criterion 2. If Criteria 1, 2 and 3 are all met, sarcopenia is considered severe.
1. Low muscle strength 2. Low muscle quantity or quality 3. Low physical performance

A study showed the prevalence of sarcopenia was 14.1% in Japanese residents aged 65 years or older (6). Sarcopenia is very prevalent in the elderly, which imposes a heavy burden on individuals and society. However, there are no clear treatments and interventions available (7). In addition, a systematic review showed that estimates of sarcopenia prevalence ranged from 9.9 to 40.4% due to significant differences in the prevalence among different populations (8). Over the past two decades, related studies on sarcopenia began to become hot. Meanwhile, many sarcopenia articles were published in all kinds of journals. However, research on global research trends in the field of sarcopenia remains vacant. Therefore, it is necessary to investigate global research trends on sarcopenia. Publications are an important indicator of the research contribution of a country or research institution (9). Bibliometric analysis can provide information based on databases to evaluate trends in quantity and quality over a certain period, which provides a way to focus on developing specific fields and to evaluate the contributions of countries, journals, and institutes (10). Therefore, this article aims to evaluate the current situation and future trends in the field of sarcopenia research.

## Materials and Methods

### Data Sources

Bibliometric analysis of the sarcopenia-related research was conducted on August 21, 2021. Sarcopenia-related articles published from 2001 to 2020 were retrieved from the PubMed database and the Web of Science online database. Science Citation Index Expanded (SCI-EXPANDED), Social Sciences Citation Index (SSCI), Emerging Sources Citation Index (ESCI), Conference Proceedings Citation Index-Social Science and Humanities (CPCI-SSH), Conference Proceedings Citation Index-Science (CPCI-S), and Arts and Humanities Citation Index (A&HCI) are all included. The impact factor (IF) of journals came from the journal citation reporting database. The data in this study are derived from a public database and no ethical issues are involved. Therefore, this study was not reviewed by the ethics committee.

### Search Strategy

In the Web of Science online database, the search terms were: topic = sarcopenia and publishing year = (2001–2020). The search results were refined by articles, review, meeting abstract, letter, and proceedings paper to identify the core literature for this bibliometric analysis. In the PubMed database, the search term “sarcopenia (Mesh Terms)” does not retrieve all the publications concerning sarcopenia. Consequently, “sarcopenia” (All Fields) and results from 2001 to 2020 can retrieve all publications concerning sarcopenia. The document types retrieved included clinical trial, meta-analysis, randomized controlled trial (RCT), review, systematic review, case reports, and basic research studies. To find basic research studies, the species was identified as an “other animal”. Using the total reference frequency, the average number of references per item, and the h-index to assess the publication quality. The quantity of literature and publication trends were analyzed based on the total amount of publication, research organization, research field, type of articles, and journals.

### Data Collection

Information of all the eligible publications, including the title, author's name and affiliation, nationality, keywords, year of publication, and the journal, was selected from the scientific online database. All the authors screened, collected, and extracted the data, resolved the differences, and reached a consensus.

### Statistical Analysis

The descriptive statistical analysis in this study was analyzed by using linear regression (Statistical significance was set at *P* < 0.05). All these were conducted by SPSS software (version 19.0, IBM Incorporation, Armonk, New York, USA).

### Visualized Analysis

The VOS viewer software enables the analysis of the bibliometric visualization of publications. In this study, the VOS viewer was used for co-occurrence, co-authorship, co-citation, and bibliographic coupling analysis.

## Results

### Description for This Study

Our study is a bibliometric analysis of publications on sarcopenia. All the data are original and based on a large number of statistics. Bibliometric analysis can provide information based on databases to evaluate trends in quantity and quality over a certain period, which provides a way to focus on developing specific fields and to evaluate the contributions of countries, journals, and institutes.

### Sarcopenia-Related Publications Growth Trends in the World

**Figure 1:**
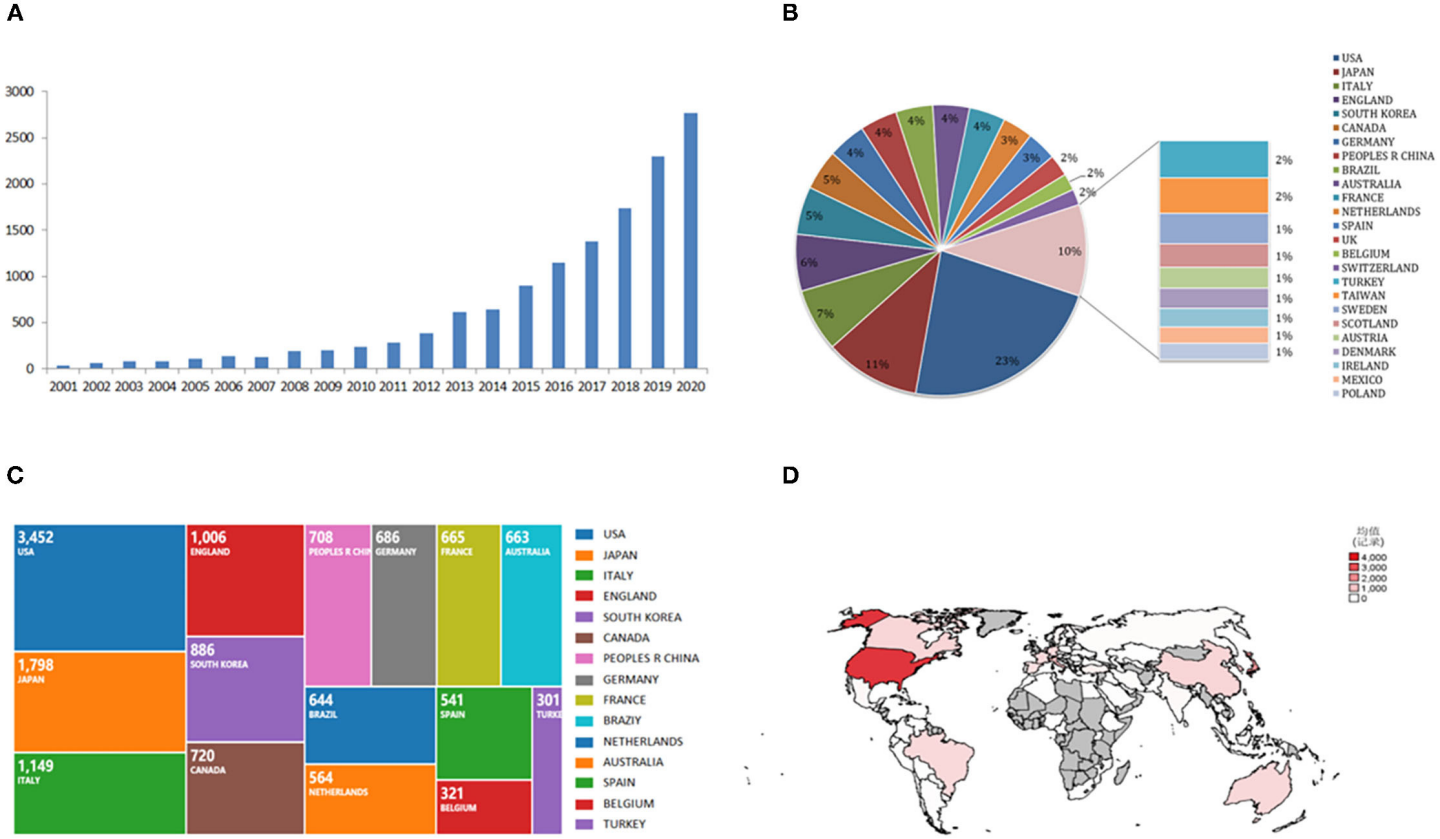
The sarcopenia-related articles globally. **(A)** A global number of sarcopenia publications per year. Blue bars indicate the number of sarcopenia articles. **(B)** Percentage of studies from the top 25 countries. **(C)** Sum of Sarcopenia-related articles from the top 15 countries over time. **(D)** Heat map showing the distribution of sarcopenia publications in the world.

Based on search criteria, there are 13,421 articles, including article (9,261), review (1,941), meeting abstract (1,778), letter (416), and others, were published in the world from 2001 to 2020 in the Web of Science. As Figure 1A shows, the global number of sarcopenia-related publications showed a rising trend year by year (*R*^2^ = 0.753, *P* < 0.01) from 39 in 2001 to 2,767 in 2020. Most research was published in 2020 (2,767, 20.617%).

There are 105 countries/regions that participated in sarcopenia-related research around the world. Among them, the largest number of sarcopenia articles are published by the USA (3,452, 25.721%), followed by Japan (1,798, 13.397%), Italy (1,149, 8.561%), England (1,006, 7.496%), and South Korea (886, 6.602%) (Figures 1B,C). The annual publication trends of sarcopenia in these countries all showed significant positive trends year by year. Figure 1D shows the distribution of sarcopenia publications in the world (Figure 1).

### Citations and H-Index Analysis of Sarcopenia-Related Publications

**Figure 2:**
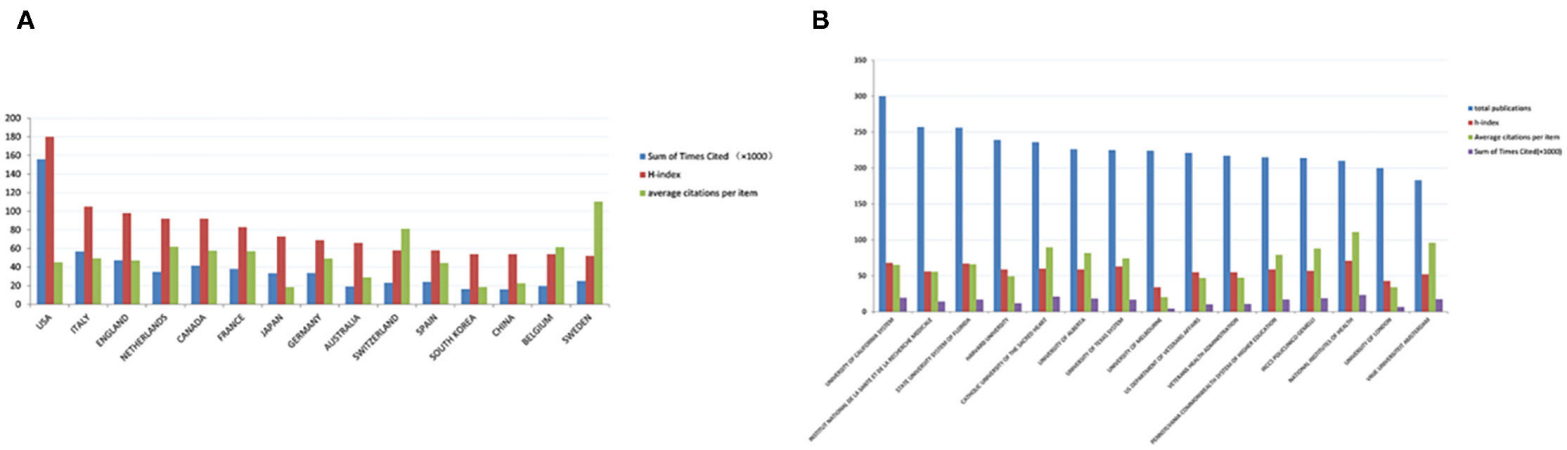
Citation and h-index analyses globally. **(A)** Sum of timed cited, h-index, and average citations per item for sarcopenia articles from the top 15 countries. **(B)** Total publications, h-index, average citations per item, and the sum of times cited for sarcopenia articles from the top 15 global institutions.

According to the data from the Web of Science, sarcopenia papers from the USA possessed the highest total citation frequencies (155,825), followed by Italy (56,829), England (47,321), and Canada (41,531). For the average citation frequencies per item, Sweden ranked first (110.48), followed by Switzerland (81.09), Scotland (73.44), Netherlands (61.85), and Belgium (61.43). In terms of the H-index, the index of the USA was 180, which was ranked first, followed by Italy with an index of 105 (Figure 2A).

A total of 7,287 institutions from different countries/regions contributed to sarcopenia-related research between 2001 and 2020. Figure 2B shows the top 15 most contributing institutions in the world. Among them, eight in the USA, Institut National de la Sante et de la Recherche Medicale and University of London were in the UK, the Catholic University of the Sacred Heart and IRCCS Policlinico Gemelli were from Italy, the University of Alberta was from Canada, the University of Melbourne was in Australia, VRIJE Universiteit Amsterdam was from the Netherlands. The largest amount of sarcopenia articles was from the University of California System, which produced 300 publications and had 19,544 total citations and 68 h-index (Figure 2).

### Difference of Research Category and Article Types of Sarcopenia-Related Publications

**Figure 3:**
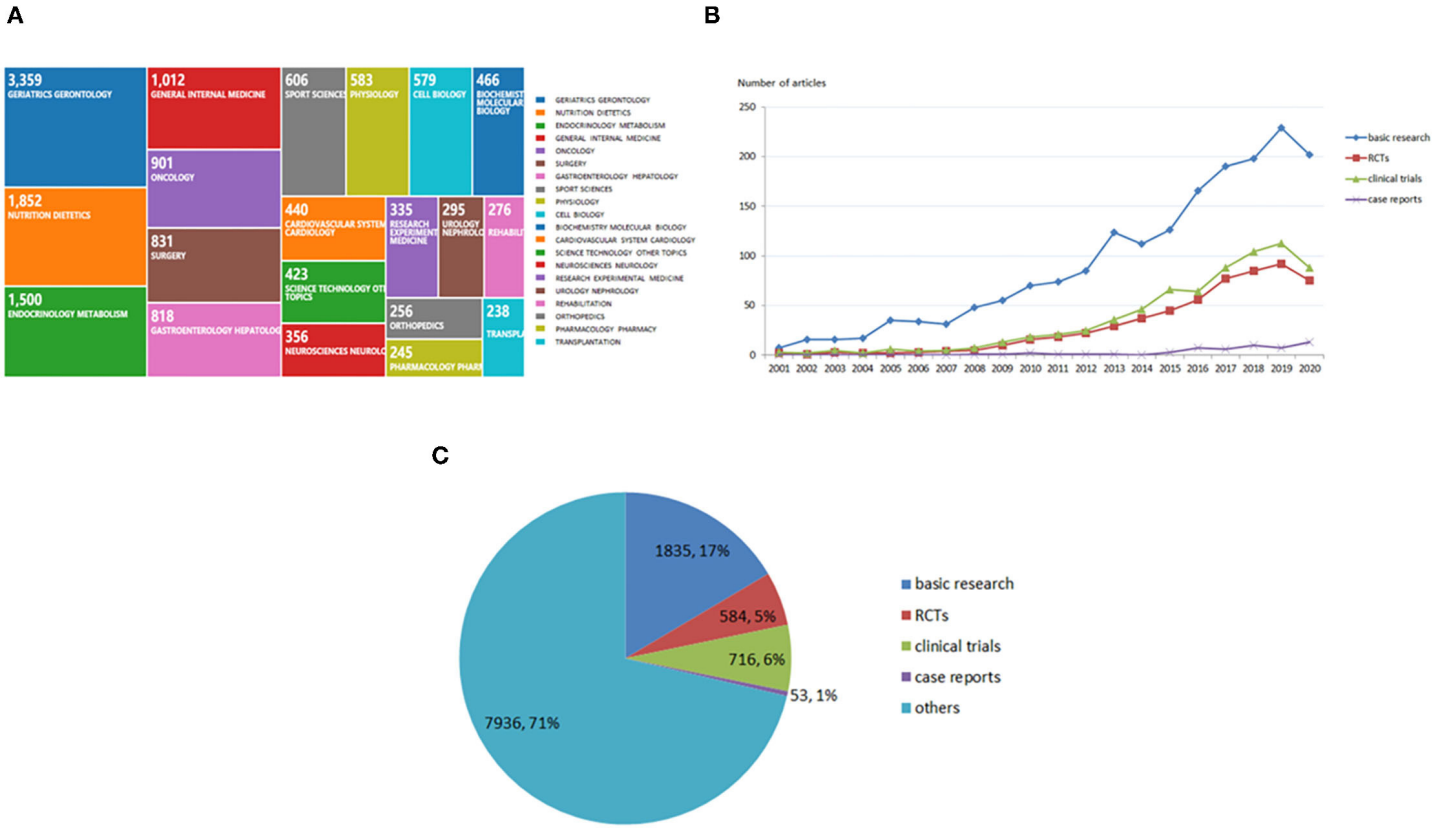
Research area and article types globally. **(A)** Research area in global sarcopenia research. **(B)** Randomized controlled trials, clinical trials, case reports, and basic research worldwide from 2001 to 2020. **(C)** Percentage of various types of articles.

Globally, sarcopenia-related publications were categorized into 74 research areas. Figure 3A shows the top 20 research areas related to sarcopenia, and the most frequently observed area was geriatrics gerontology with 3,359 articles (25.020%), followed by nutrition dietetics with 1,852 articles (13.792%), endocrinology metabolism with 1,500 articles (11.177%), general internal medicine with 1,012 articles (7.526%), and oncology with 901 articles (6.706%).

According to the PubMed database, basic research was popular among researchers. There are 1,835 basic research articles in the past 20 years, accounting for 16.496% of the total sarcopenia articles. Additionally, 716 clinical trials (6.437%), 584 RCTs (5.250%), and 53 case reports (0.476%) were published in the sarcopenia field (Figure 3).

### Co-occurrence Analysis

**Figure 4:**
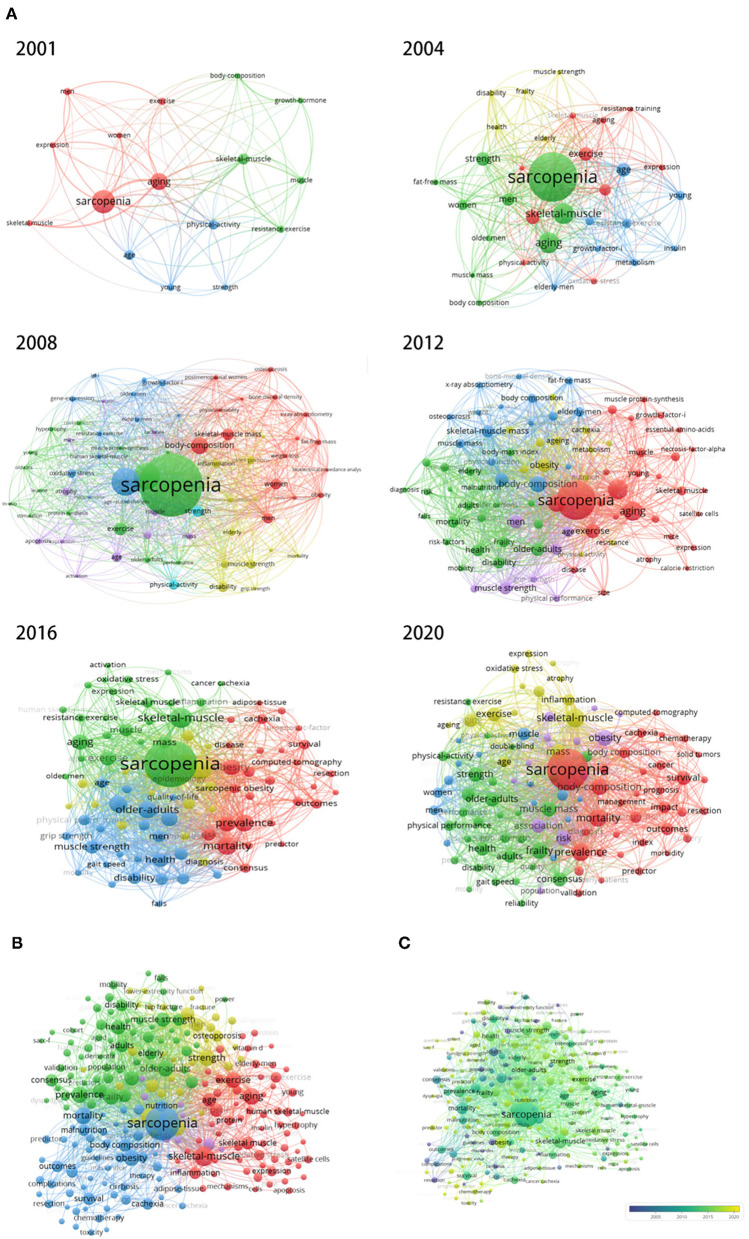
Keyword maps globally. **(A)** Global keyword maps in different years. **(B)** Keyword maps in the past 20 years. In this visualized network, each keyword is represented by a node. Node size reflects the number of publications in the node, and the distance between two nodes indicates the connectivity of the nodes as determined by co-occurrences. The larger the number of publications for which two nodes are both found, the stronger the relationship between the nodes. VOS viewer has its clustering technique based on citation relations between clusters. Colors represent groups of nodes that are relatively strongly related to each other. **(C)** Distribution of keywords according to their time of appearance. Keywords in blue appeared earlier than those in yellow.

Co-occurrence analysis can be used to discover directions and popular topics of specific areas, which is vital for monitoring the development of science and programs. From the global keyword's maps, the co-occurrence networks of keywords for 2001, 2004, 2008, 2012, 2016, and 2020 are shown in Figure 4A. Finally, a global keyword map is made using keywords in the past 20 years (Figures 4B,C). In Figure 4C, the keyword, such as “obesity”, “body composition”, and “nutrition”, showed that the body composition and nutritional intervention may be a hot research field, “prevalence”, “old adults”, and “mortality” may indicate that epidemiological studies of sarcopenia are also popular around the world, and “inflammation” and “satellite cell” may be indicated that basic research on sarcopenia is a research focus. From Figure 4, we can see the number of global keywords regarding sarcopenia showed a rising trend from 280 in 2001 to 7,057 in 2020 (Figure 4).

As can be seen from Table 3, the keywords of the literature in the world on sarcopenia are mainly sarcopenia, aging, skeletal muscle, strength, and body composition (Table 3).

**Table 3:** The number of keywords and the top five in the world.

**Year**	**The number of keywords**	**The top five**
2001	280	Aging, sarcopenia, skeletal-muscle, young, physical- activity
2004	553	Sarcopenia, aging, skeletal-muscle, exercise, body-composition
2008	1,081	Sarcopenia, aging, skeletal-muscle, body composition, strength
2012	1,712	Sarcopenia, aging, skeletal-muscle, strength, body-composition,
2016	3,650	Sarcopenia, body-composition, skeletal-muscle, mortality, older-adults
2020	7,057	Sarcopenia, mortality, prevalence, skeletal-muscle, body-composition

### Bibliographic Coupling Analysis

#### Journal

**Figure 5:**
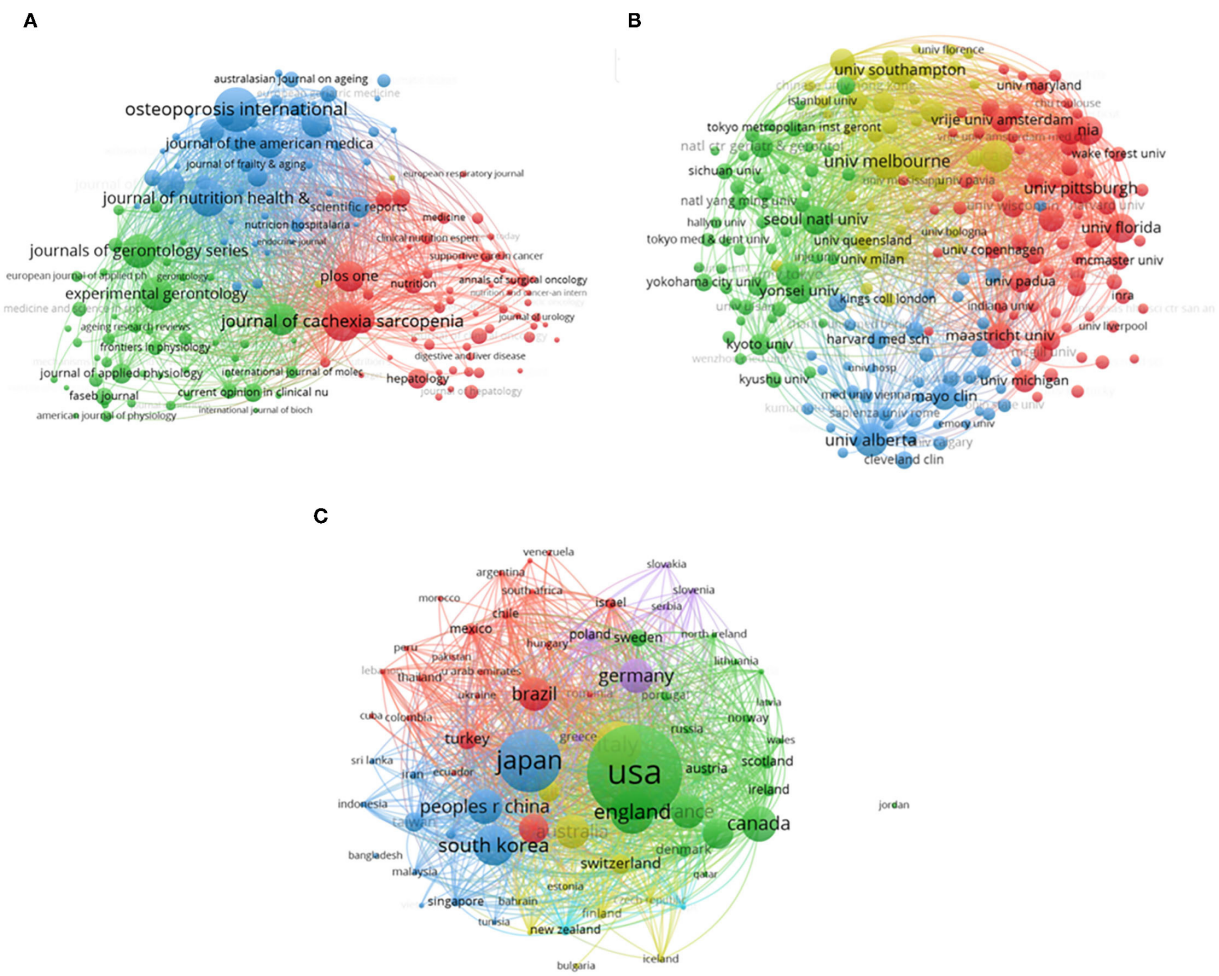
Bibliographic coupling analysis of global research about sarcopenia. **(A)** Mapping of the 156 identified journals on sarcopenia. **(B)** Mapping of the 203 institutions on sarcopenia. **(C)** Mapping of the 74 countries on sarcopenia. The line between two points in the figure represents that two journals/institutions/countries had established a similar relationship. The thicker the line is, the closer the link between the two journals/ institutions/countries is.

Bibliographic coupling analysis is a method that uses citation analysis to establish a similarity relationship between different documents. The name of journals in total publications was analyzed by VOS viewer. Figure 5A shows that 156 journals (Minimum number of documents of a source: 15) appeared in total link strength. Journal of Cachexia Sarcopenia and Muscle (total link strength = 813,939 times), Journal of Nutrition Health and Aging (total link strength = 800,175 times), Journal of the American Medical Directors Association (total link strength = 712,116 times), Journals of Gerontology Series A-Biological Sciences and Medical (total link strength = 612,559 times) and Nutrients (total link strength = 587,869 times) were the top five journals with large total link strength.

#### Institution

In total, 203 institutions (Minimum number of documents of an organization: 30) were analyzed in this study (Figure 5B). The University of Melbourne (total link strength = 945,327 times) ranked first, followed by University Cattolica Sacro Cuore (total link strength = 945,214 times), the University of Alberta (total link strength = 795,254 times), NIA (total link strength = 794,788 times), and Seoul National University (total link strength = 777,215 times).

#### Country

There are 74 countries (Minimum number of documents of a country: five) that were analyzed in this study (Figure 5C). The USA was the NO.1 with a total link strength of 11,675,553 times, followed by Japan (total link strength = 7,494,135 times), Italy (total link strength = 5,754,482 times), England (total link strength = 5,041,102 times), and South Korea (total link strength = 4,482,793 time) (Figure 5).

### Co-citation

#### Publication

**Figure 6:**
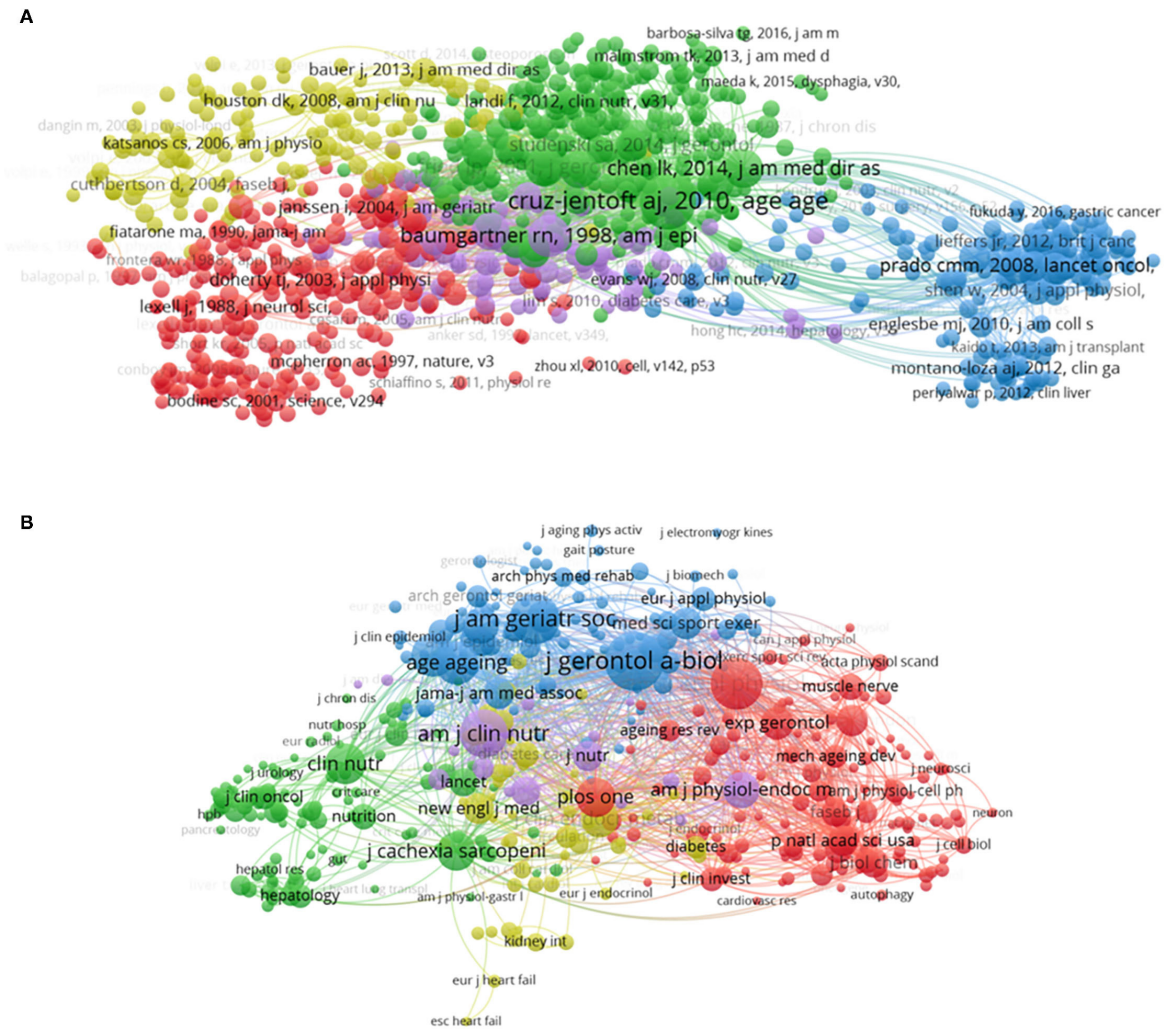
Mapping of co-citation on sarcopenia. **(A)** Mapping of co-cited references (The 924 points with different colors represent the 924 cited references. The size of the points represents the citation frequency. A line between two points means that both were cited in one paper. A shorter line indicates a closer link between the two papers. Points in the same color belong to the same research direction). **(B)** Mapping of co-cited journals (The 409 points with different colors represent the 409 identified journals. The size of the points represents the citation frequency. A line between two points means that both were cited in one journal. A shorter line indicates a closer link between the two journals. Points in the same color belong to the same research direction).

Correlations between items can be identified by the co-citation analysis by the number of times they were cited together. In total, 924 references (Mini number of citations: 50) are analyzed in our study (Figure 6A). Cruz-jentoft (3), (total link strength = 46,522 times); Baumgartner (11), (total link strength = 25,036 times); Jansen (12), (total link strength = 17,885 times); Fielding (13), (total link strength = 17,819 times), and Fried (14), (total link strength = 14,407 times) were the top five studies with large total link strength.

#### Journal

In total, 409 journals (mini number of citations of a source: 200) are analyzed as shown in Figure 6B. The top five journals with large total link strength were J Gerontol A-Biol (total link strength = 1,110,397 times), J Appl Physiol (total link strength = 936,114 times), J Am Geriatr Soc (total link strength = 712,455 times), Am J Clin Nutr (total link strength = 692,601 times), and J Clin Endocr Metab (total link strength = 5,43,281 times).

### Co-authorship Analysis

#### Author

**Figure 7:**
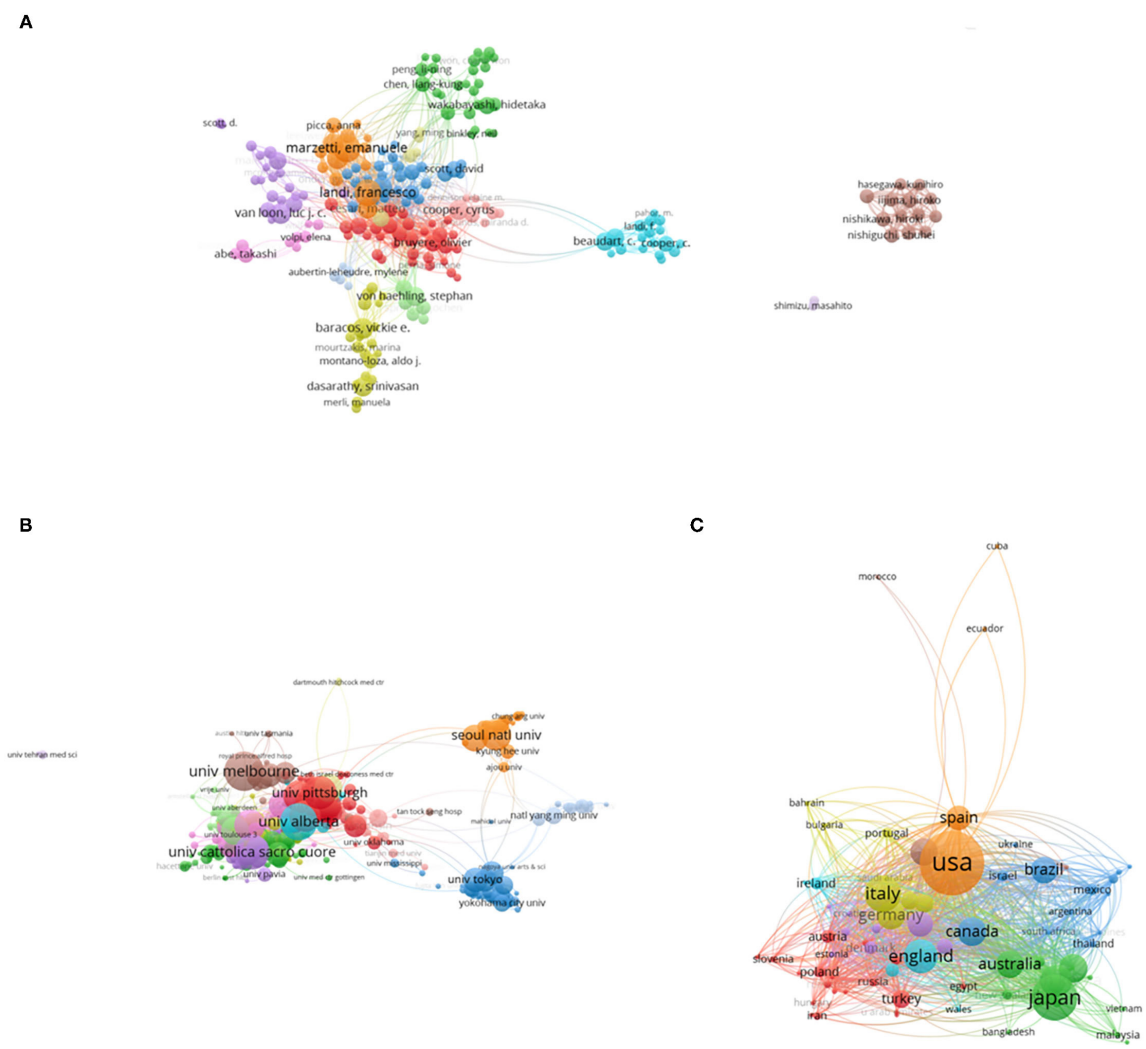
Co-authorship analysis of global research about sarcopenia. **(A)** Mapping of the 245 authors co-authorship analysis on sarcopenia. **(B)** Mapping of the 461 institutions co-authorship analysis on sarcopenia. **(C)** Mapping of the 74 countries co-authorship analysis on sarcopenia. The size of the points represents the co-authorship frequency. The line between two points in the figure represents that two authors/institutions/countries had established collaboration. The thicker the line is, the closer the collaboration between the two authors/institutions/countries is.

Co-authorship analysis judged the relevance of the items by the number of its co-authored papers. In total, 245 authors (Minimum number of documents of an author: 15) were analyzed in our study (Figure 7A). The top five authors with large total link strength were Landi, Francesco (total link strength = 464 times), Nishiguchi, Shuhei (total link strength = 380 times), Nishikawa, Hiroki (total link strength = 380 times), Enomoto, Hiroyuki (total link strength = 378 times), and Iijima, Hiroko (total link strength = 378 times).

#### Institution

In Figure 7B, 461 institutions (minimum number of documents of an organization: 15) were analyzed. The top five institutions were Univ Pittsburgh (total link strength = 703 times), National Institute on Aging (NIA) (total link strength = 565 times), Univ Southampton (total link strength = 533 times), Tufts Univ (total link strength = 503 times), and Univ Clif San Francisco (total link strength = 481 times).

#### Country

Publications (defined as the minimum number of documents of a country that were used more than five) identified in the 74 countries were analyzed by VOS viewer (Figure 7C). The top five countries were the following: USA (total link strength = 2015 times), England (total link strength = 1,742 times), Italy (total link strength = 1,371 times), Germany (total link strength = 1,033 times), and France (total link strength = 978 times) (Figure 7).

#### High Contribution Journals in the Sarcopenia Field and Funding Agencies in the World

**Figure 8:**
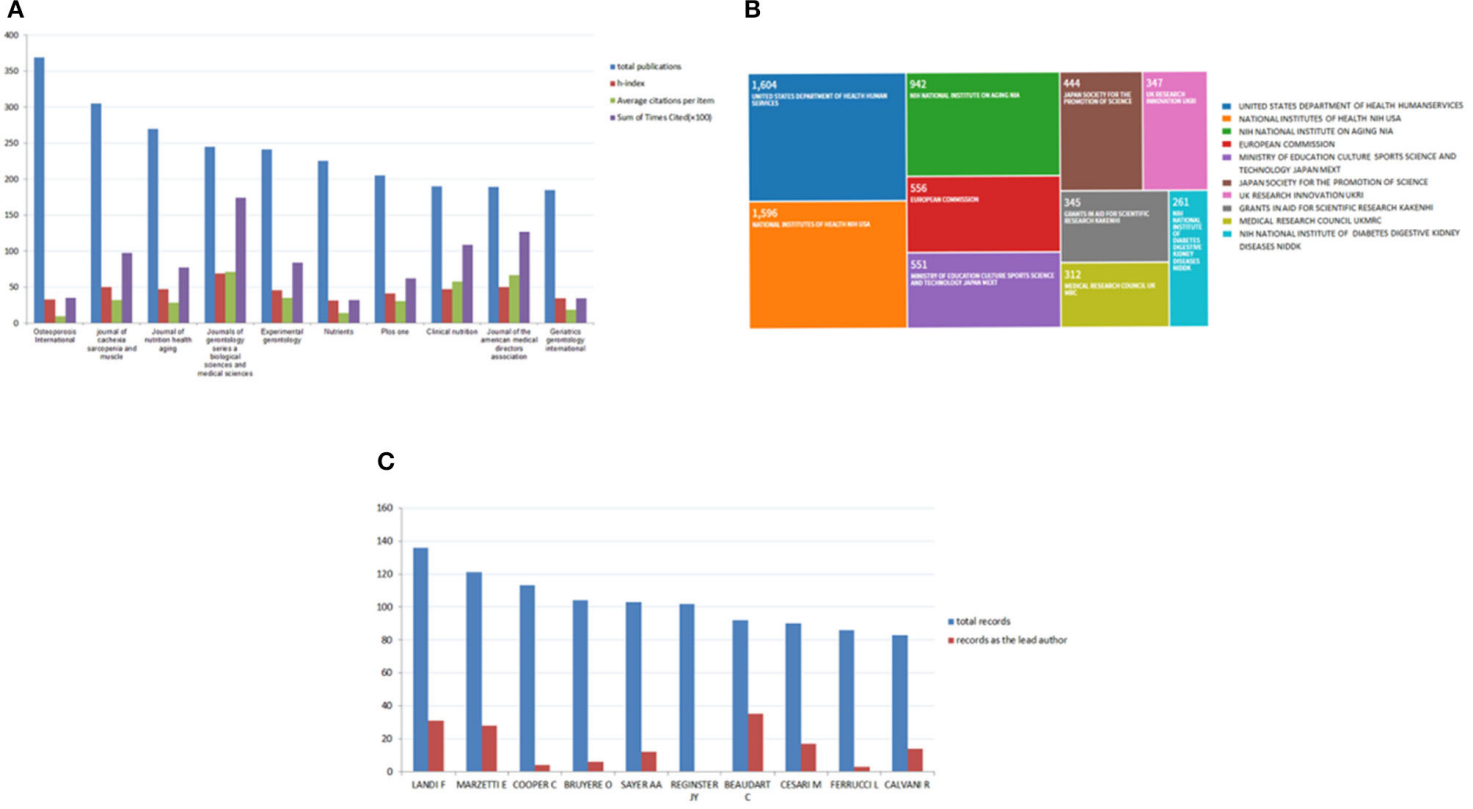
**(A)** The top 10 journals with the highest contribution in the sarcopenia field. **(B)** High contribution funding agency in the world. **(C)** The top 10 authors in the world.

A total of 2,019 journals published sarcopenia articles. Figure 8A shows the 10 journals publishing the largest number of publications. In the sarcopenia field, Osteoporosis International is the most contribution journal in the world with the largest number of publications. In the world, a total of 6,557 funding agencies support sarcopenia-related research. Among them, the United States Department of Health Human Services was the main funding organization, which has 1,604 records, ranking first (Figure 8B).

#### High Impact Authors in the World

Figure 8C shows the top 10 authors with the highest impact. About 1,030 publications were from the top 10 authors, which accounts for 7.675% and the top 10 authors published a total of 150 articles as the lead author. Among them, the highest impact author is LANDI F with 136 records, followed by MARZETTI E with 121 records (Figure 8).

#### Ten Most-Cited Articles on Sarcopenia in the World

Table 4 shows the top 10 articles. The number of citations for the top 10 articles ranged from 1,044 to 5,701 in the world. The top 10 articles in the world were published from 2002 to 2019 (Table 4).

**Table 4:** The Top 10 most frequently cited articles on sarcopenia in the world.

**Title**	**The first author**	**Journal**	**Year**	**Type**	**Times cited**	**IF**
Sarcopenia: European consensus on definition and diagnosis	Cruz-Jentoft, AJ	Age and aging	2010	Article	5,701	10.662
Definition and classification of cancer cachexia: an international consensus	Fearon, K	Lancet oncology	2011	Review	2,248	41.313
Sarcopenia: revised European consensus on definition and diagnosis	Cruz-Jentoft, AJ	Age and aging	2019	Article	2,066	10.662
Low relative skeletal muscle mass (sarcopenia) in older persons is associated with functional impairment and physical disability	Janssen, I	Journal of the American geriatrics society	2002	Article	1,782	5.560
Sarcopenia in Asia: consensus report of the asian working group for sarcopenia	Chen, LK	Journal of the American medical directors association	2014	Review	1,750	4.661
Sarcopenia: an undiagnosed condition in older adults. current consensus definition: prevalence, etiology, and consequences. international working group on sarcopenia	Fielding, RA	Journal of the American medical directors association	2011	Article	1,556	4.664
Prevalence and clinical implications of sarcopenic obesity in patients with solid tumors of the respiratory and gastrointestinal tracts: a population-based study	Prado, CMM	Lancet oncology	2008	Article	1,440	41.313
Cancer cachexia in the age of obesity: skeletal muscle depletion is a powerful prognostic factor, independent of body mass index	Martin, L	Journal of clinical oncology	2013	Article	1,091	44.544
Age-associated changes in skeletal muscles and their effect on mobility: an operational diagnosis of sarcopenia	Lauretani, F	Journal of applied physiology	2003	Article	1,073	3.533
Aging and sarcopenia	Doherty, TJ	Journal of applied physiology	2003	Review	1,044	3.532

## Discussion

### Global Research in the Sarcopenia-Related Field

The number of articles published has increased significantly over the years, which has made keywords more prevalent. Studies through Bibliometric and visualized research can be used to show the status of research in a certain field and to predict future trends. As the global population ages, the prevalence of sarcopenia will increase. Therefore, there will be more institutions dedicated to sarcopenia research. Additionally, more studies on sarcopenia will be published in the next few years.

By the co-occurrence analysis, we found that directions and research focus in this field. The keywords as the most important part of articles were used to create a co-occurrence network map. From Figure 4B, all the keywords from 2001 to 2020 were divided into three clusters: individual study, cohort study, and study revealing the link between sarcopenia and other diseases. In the center of the co-occurrence map, the keywords included sarcopenia, nutrition, skeletal muscle, aging, and body composition, which revealed the hot spot of research in this field in the past 20 years. On the other hand, overlay visualization was also important for monitoring the directions of research (15). In the overlay visualization map (Figure 4C), colors indicate publication years. The blue color means that published early and yellow-colored keywords mean that published later. We observed keywords such as cancer, cirrhosis, and cachexia appear in recent publications, which showed the link between sarcopenia and other diseases will be the next research hotspot. At the same time, some studies will delve deeper into molecular mechanisms (16).

### Status of Global Publications

The total citations and h-index of countries represent their academic influence and publication quality. The USA contributed the most to global sarcopenia-related research with high total publications and total citations, especially the total citations of the USA were far was ahead of other countries. As a result, the US can be considered a leader in this field. Osteoporosis International published the largest number of publications, which is the most contribution journal in the world. Of 10 journals publishing the largest number of reports, three journals are from the UK, two journals are from the USA, and the rest are from Germany, France, Switzerland, Scotland, and Australia. The journals on the list may be included the main discoveries in this field in the future. Institutes from the top five countries were the leader in sarcopenia-related research. All of the top 15 institutes were located in the top nine countries. The United States Department of Health Human Services and the National Institutes of Health were the main funding organization, whose contribution was far more than any others. The top 10 authors who published most articles in sarcopenia were also listed and we can keep track of the scientific frontiers of this field by paying close attention to their published work. At present, the research on sarcopenia is mainly focused on geriatrics gerontology and basic research. In this study, a similar relationship between publications in terms of journal, institution, and country was established through bibliographic coupling analysis. Bibliographic coupling occurs when two works cite a common third work in their articles (17). The data from this study indicated that the most related journal is the Journal of Cachexia Sarcopenia and Muscle and the University of Melbourne was the leading Institution. The USA is the leader in this research field. The degree of collaboration and collaboration between authors, institutions, and countries was assessed by co-authorship analysis. In general, the higher the overall link strength, the closer the authors/institutions/countries cooperate with other countries. Co-citation analysis can investigate the impact of studies by calculating the number of studies cited together.

### Limitations

This study had some limitations. There are some limitations on the methodological quality of our study, Future improvements in methodology are necessary. First, bibliometric analysis can only measure the quality of scientific research through the influence index of literature. However, there is no absolute equivalence between them. For example, a highly cited publication does not mean high scientific quality. Second, the data in our study were from the PubMed database and the Web of Science online database. Therefore, we may omit some other literature. Third, we only selected literature written in English as the research objects, excluding many non-English publications. Finally, some recent research of high scientific quality might not be emphasized due to fewer citations. Therefore, paying attention to the latest publications is also necessary.

### Future Studies Trends of the Sarcopenia Field

As the research field of sarcopenia becomes a hotspot, the future research trend of sarcopenia will receive researchers' extensive attention. Sarcopenia, obesity, and sarcopenic obesity are vital characteristics of the aging process that can lead to important health problems. Future studies may focus on the interrelationships between these changes in body composition (18). Malnutrition plays a key role in the pathogenesis of sarcopenia. The quality of diet throughout the life cycle is closely related to its incidence, and nutritional interventions may be able to reduce or reverse the pathogenesis of sarcopenia. The effects of energy and protein intake or other key nutrients on muscle may also be a hot direction for future research (19). Exercise training, especially resistance training, has long been recognized as the most promising way to increase muscle quality and strength in older adults. Further studies of the role of exercise training in preventing muscle atrophy, promoting muscle growth, and maintaining muscle function are also hot (20). Basic research on sarcopenia and exploration of its pathogenesis is also an important research focus in the future.

## Conclusion

Global sarcopenia research increased rapidly from 2001 to 2020, especially in recent years. The USA was the leader of sarcopenia-related research. The study of sarcopenia will continue to focus on aging, nutrition, exercise, and delve deeper into molecular mechanisms in the future. In addition, revealing the link between sarcopenia and other diseases may be the next research hotspot.

## Data Availability Statement

The original contributions presented in the study are included in the article/supplementary material, further inquiries can be directed to the corresponding author/s.

## Author Contributions

DY and HJ participated in study design, data collection, statistical analysis, and manuscript preparation. QL, JZ, and BM participated in the data check. WX and YL conceived and revised this article. All authors approved the final version of the manuscript.

## Funding

This study was supported by the National Natural Science Foundation of China (Nos. 81874030 and 82072506), Science and Technology Innovation Program of Hunan Province (No. 2021RC3025), Provincial Natural Science Foundation of Hunan (No. 2020JJ3060), Provincial Clinical Medical Technology Innovation Project of Hunan (No. 2020SK53709), Innovation-Driven Project of Central South University (No. 2020CX045), Wu Jieping Medical Foundation (No. 320.6750.2020-03-14), National Clinical Research Center for Geriatric Disorders (Xiangya Hospital, No. 2021KFJJ02), National Clinical Research Center for Orthopedics, Sports Medicine and Rehabilitation (No. 2021-NCRC-CXJJ-PY-40), Exploration and Innovation Project for Undergraduate Students of Hunan (Nos. S2021105331047 and S2021105331107), and Central South University (No. XCX2021046).

## Conflict of Interest

The authors declare that the research was conducted in the absence of any commercial or financial relationships that could be construed as a potential conflict of interest.

## Publisher's Note

All claims expressed in this article are solely those of the authors and do not necessarily represent those of their affiliated organizations, or those of the publisher, the editors and the reviewers. Any product that may be evaluated in this article, or claim that may be made by its manufacturer, is not guaranteed or endorsed by the publisher.
